# A Challenging “Achy” Neck

**DOI:** 10.7759/cureus.24544

**Published:** 2022-04-27

**Authors:** John P Yaro, Yousef Ibrahim, Amandeep Mann, Jo-Yen Chan, Mohamed-Shaji Mansuri

**Affiliations:** 1 Otolaryngology, University Hospitals of Derby and Burton, Derby, GBR; 2 Pathology, University Hospitals of Derby and Burton, Derby, GBR; 3 Radiology, University Hospitals of Derby and Burton, Derby, GBR

**Keywords:** liposarcoma, rare diseases, posterior neck, angiolipoma, giant lipoma

## Abstract

Lipomas are common benign mesenchymal tumours that may occur in many regions of the body. Giant neck lipomas are uncommon, especially when they arise from the neck and extend into the thorax. In this case report, we present a unique case of a giant submuscular lipoma involving the posterior neck triangle extending down to the scapular tip. A 43-year-old male presented with a six-month history of two slow-growing masses involving the left neck and scapular region. MRI demonstrated a single large fat suppressing lesion underlying the left trapezius muscle extending down to the scapula with homogenous signal return and smooth outline measuring 4.5x7.5 cm by 16 cm. Histology showed features consistent with lipoma. Giant lipomas in the neck post a significant diagnostic and surgical challenge. The importance of pre-operative planning and patient involvement in decision-making are essential.

## Introduction

Lipomas are common benign mesenchymal tumours that may occur in many regions of the body [1]. With an annual incidence of 1 in 1000, they account for around 50% of soft tissue neoplastic pathology [2]. Lipomas present as mobile, soft, and non-tender masses and are usually diagnosed clinically [3].

Neck lipomas are uncommon, occurring in about 13% of cases [4,5]. Of these, around 80-90% are usually found in the posterior neck triangle [4,5]. However, when they do present they are usually small lesions that are less than 5 cm in size [1]. Although patients are often asymptomatic with neck lipomas, some may present with dyspnoeic symptoms, especially when these are located in the anterior neck triangle [1].

Giant lipomas, defined as being at least 10 cm in one diameter or carrying a weight of at least 1000 grams, are exceedingly rare [1,6]. Furthermore, their presence in the posterior triangle has only ever been noted on a few occasions [7]. These usually pose both a diagnostic and surgical challenge. In this case report, we present a unique case of a giant submuscular lipoma involving the posterior neck triangle extending down to the scapular tip.

## Case presentation

A 43-year-old male presented with a six-month history of two slow-growing masses involving the left neck and scapular region. His main complaint was that of a constant dull ache. He did not have any aerodigestive or respiratory symptoms and was not overly concerned regarding the cosmesis of the lesion. Examination revealed a large posterior neck lymph node level Vb mass measuring 4.5 cm and a seemingly separate 5x5 cm mass around the left scapula tip. Both were non-tender and pseudo-fluctuant in nature without any transillumination.

**Figure 1:**
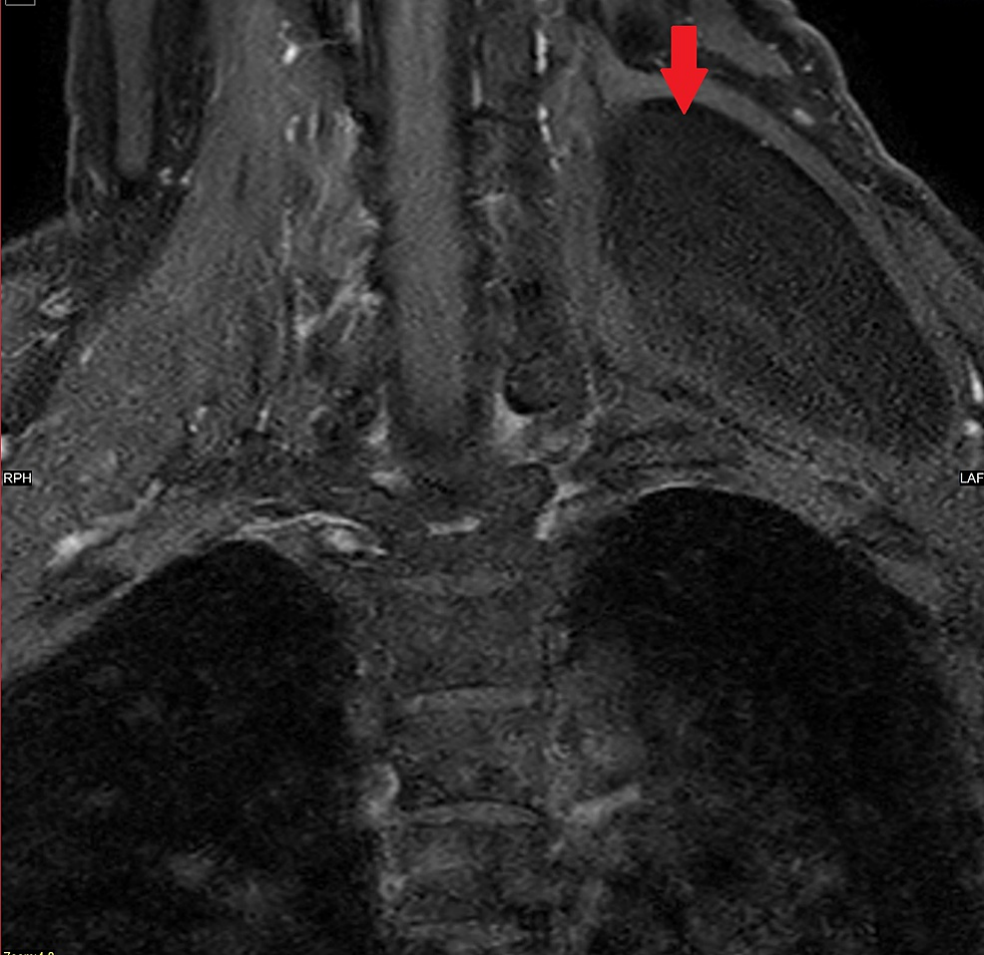
MRI Scan (Coronal)

**Figure 2:**
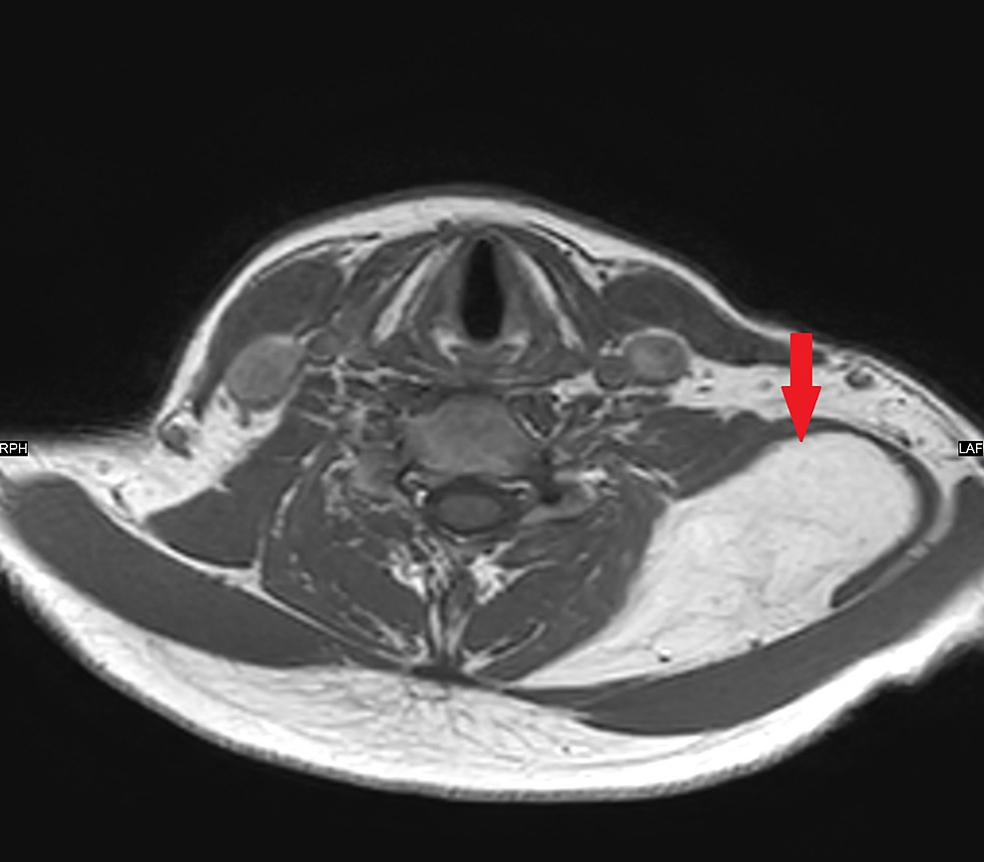
MRI Scan (Axial)

**Figure 3:**
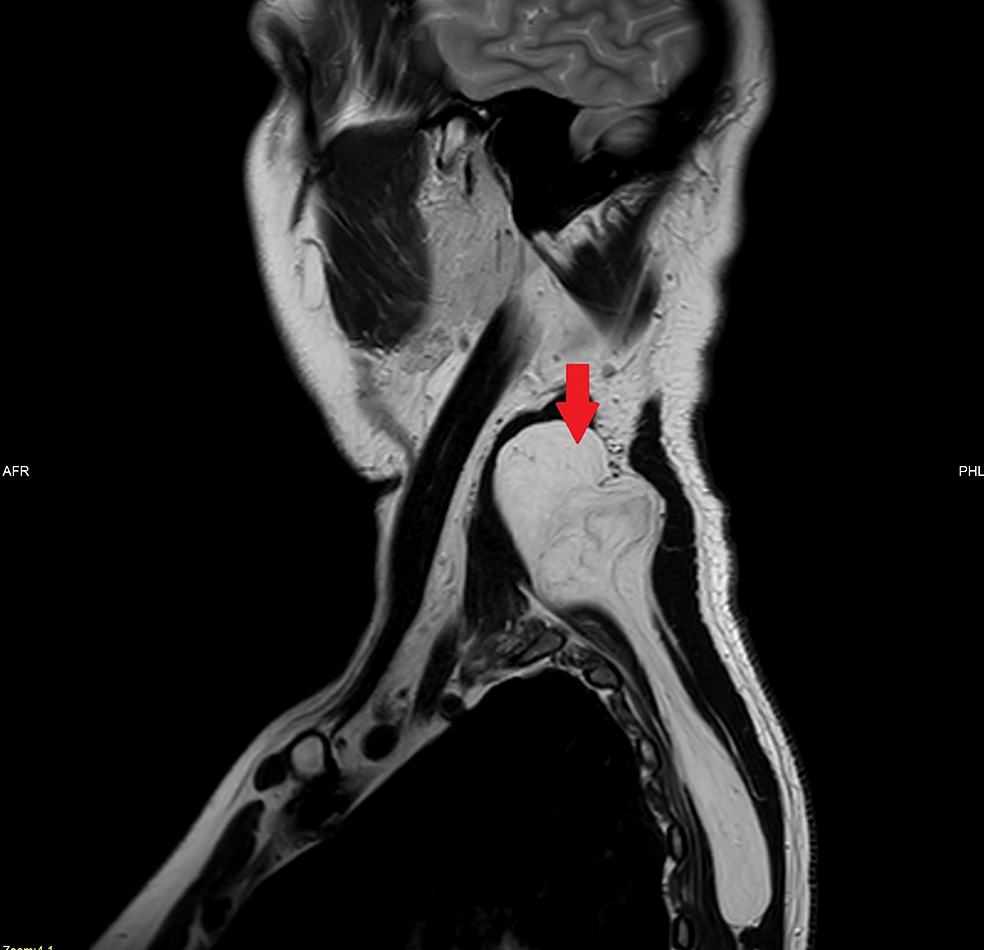
MRI Scan (Sagittal)

An ultrasound scan demonstrated two masses. One arising from the left supraclavicular fossa region into the left posterior neck, measuring 7.5x1.4x4 cm. The other lesion was overlying the left scapular and measured 6.7x1x5.3 cm. Both had appearances of a benign lipoma. Due to the size of the lipomas, the patient was sent for magnetic resonance imaging (MRI). This demonstrated a single large fat suppressing lesion underlying the left trapezius muscle extending down to the scapula with homogenous signal return and smooth outline. It measured 4.5x7.5 cm (axial); 16 cm (craniocaudal) with no adenopathy and no convincing sarcomatous changes (Figures 1-3).

**Figure 4:**
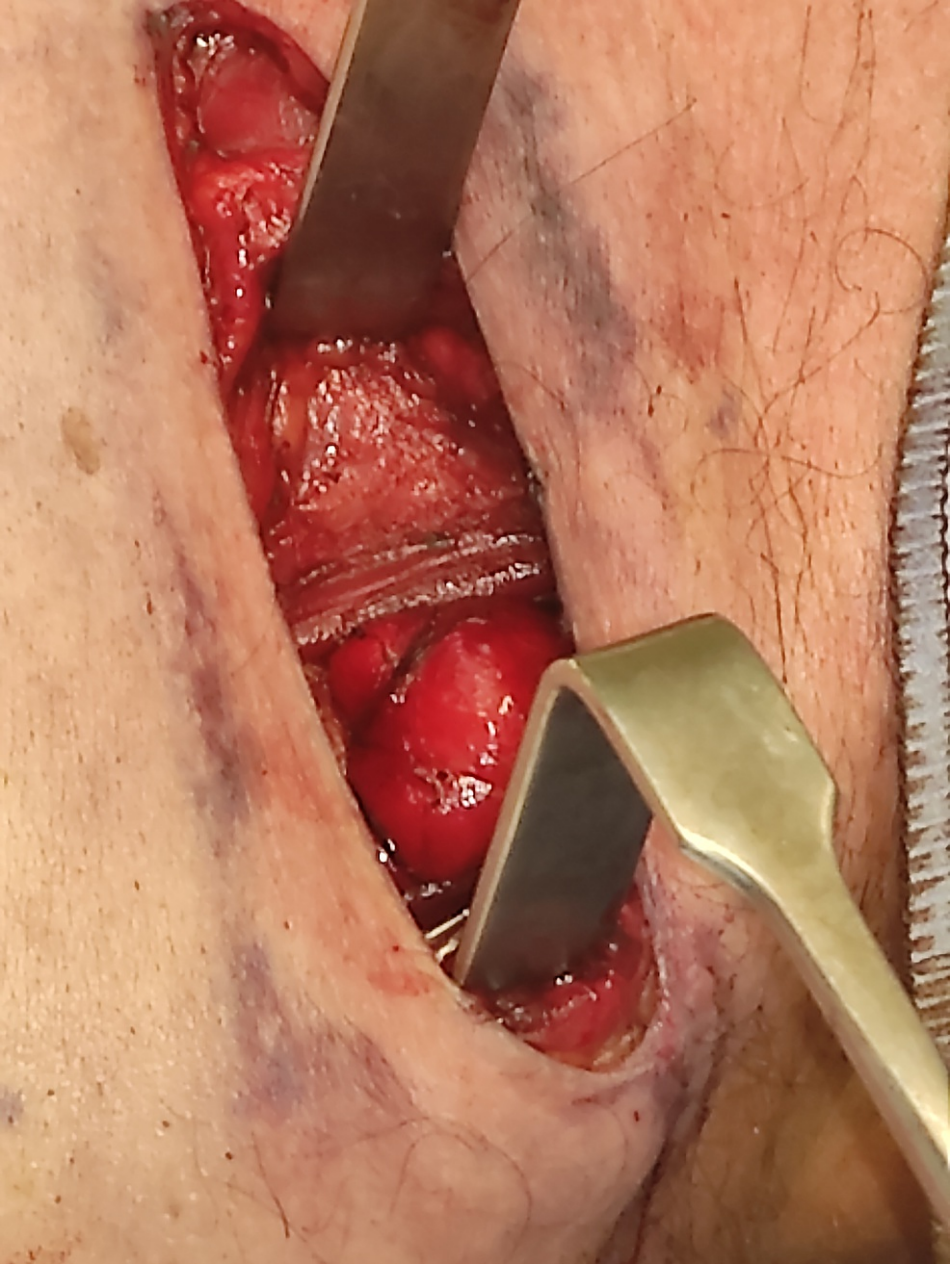
Intra-operative photo

**Figure 5:**
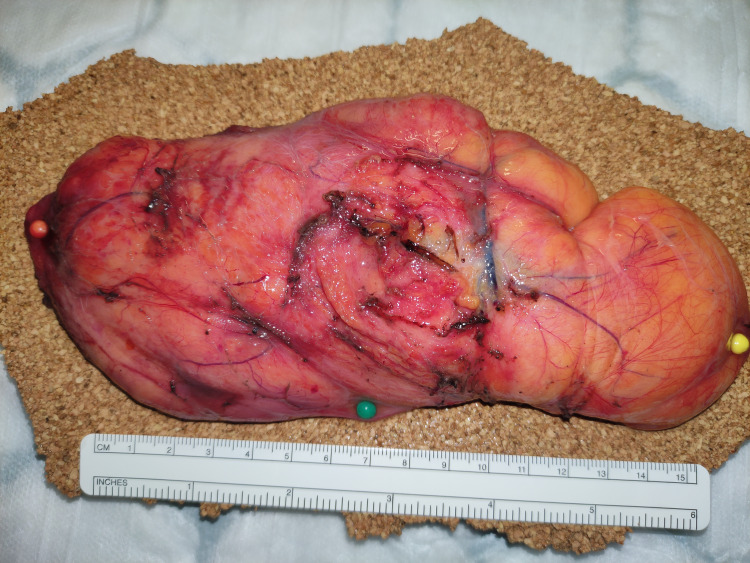
Intra-operative photos (after removal)

Complete surgical excision was carried out via a left lateral transverse skin crease neck incision. The approach began superiorly by separating the fibres of the levator scapulae and dissecting along the submuscular plane down to the scapula tip. Intra-operative photos were taken demonstrating the specimen before (Figure 4) and after removal (Figure 5). Two drains were inserted to close the residual space. The patient was discharged three days post-operatively after an uneventful recovery with a follow-up in four weeks after discussion at the multidisciplinary meeting. He was discharged at this appointment.

**Figure 6:**
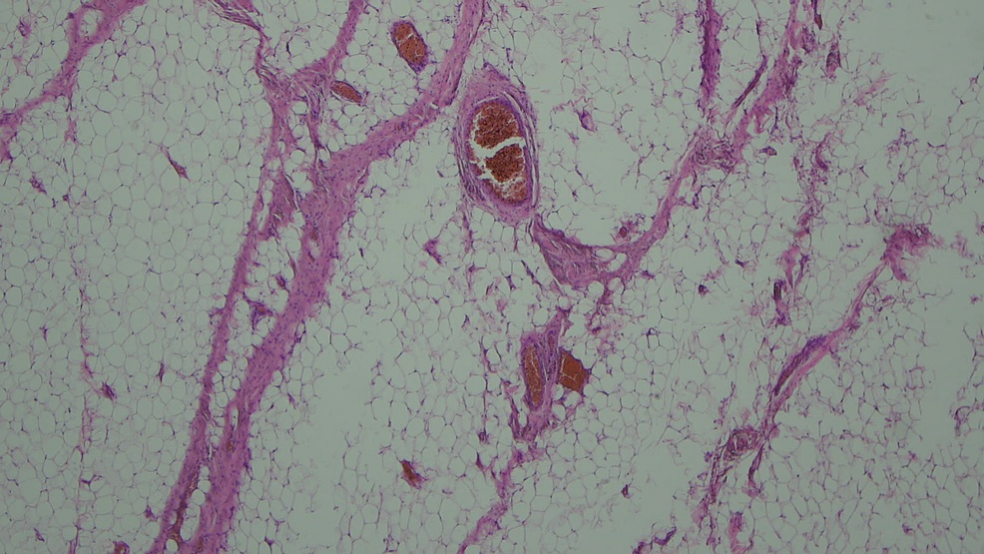
Histopathological image showing a lipoma (2 vessels present) Hematoxylin and Eosin (H&E) stain, Magnification x2

**Figure 7:**
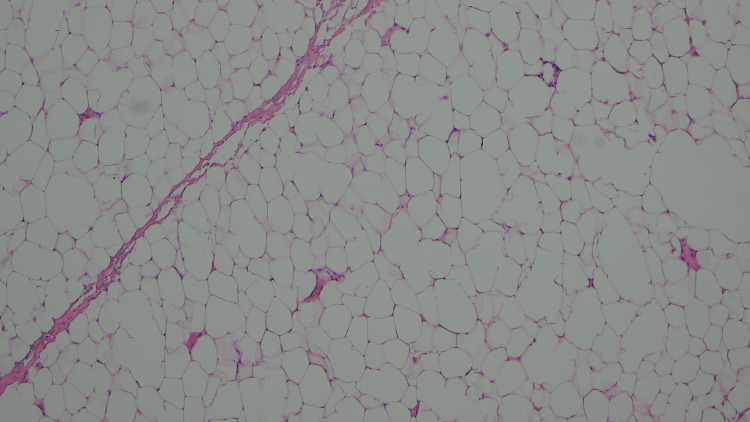
Lipoma (adipocytes) Hematoxylin and Eosin (H&E) stain, Magnification x4

The specimen was sent for histology. Macroscopic histological findings demonstrated an encapsulated fatty lesion measuring 190 mm SI (superior to inferior) x 78 mm AP (anterior to posterior) x 27 mm SD (superficial to deep). Microscopic analysis showed a likely benign lipoma but with small frequent small calibre vessels; some containing thrombi (Figures 6-7).

## Discussion

To our knowledge, there are a few reported cases in the literature involving giant lipomas of the posterior neck [7]. We have presented a case of a giant lipoma, arising from the posterior neck and extending down to the scapula tip. This was removed via a left lateral transverse skin crease neck incision. An extracapsular dissection was carried out. After identifying the capsule in the neck, this was followed into the subcapsular plane. Histology demonstrated features suggestive of lipoma.

Giant lipomas cause significant diagnostic challenges. It is essential to exclude malignancy in the form of liposarcoma as these lesions require much more extensive resection [1,8]. These would certainly pose significant difficulty in the head and neck region. Case series looking at giant lipomas in the lower limb and upper extremity revealed sarcomas rates of 8.3 to 12.5% [9,10]. In the United Kingdom, guidelines for the management of soft tissue sarcomas suggest that any lump that is increasing in size, has a size of more than 5 cm, or is painful should be referred for an urgent ultrasound scan or referred directly to a sarcoma diagnostic centre [11]. In our case, a referral to the sarcoma multidisciplinary team was not necessary as there were no features of sarcoma either on the imaging obtained or the post-operative histology. Furthermore, any patients with a suspicious ultrasound or clinical features should be referred for an MRI scan [11]. Imaging in the form of computed tomography (CT) and MRI are valuable in accurately differentiating these lesions [8]. Imaging features suggestive of malignancy include the presence of thick septa, decreased percentage of fat composition and the presence of non-adipose areas [12,13]. In some cases it may be sensible to perform a biopsy in order to guide diagnosis and subsequent management [14].

Giant lipomas in the head and neck region, due to their size, also pose a surgical challenge. Due to pressure effects on nearby structures, the normal neck anatomy may be distorted which may displace important neurovascular structures [1,15]. Patients should be well informed about these risks during the consenting process and should be involved in the decision-making process.

A variety of lipomatous tumours have been described in the literature. Benign lesions such as lipoma, angiolipoma, spindle cell lipoma, and hibernoma present around the age of 40 and are treated with simple excision in most cases [8]. Other benign tumours such as lipoblastoma and lipomatosis of nerves present in children and can be more challenging to manage [8]. Liposarcomas are malignant tumours that present in adulthood and require management in specialist centres which may involve multimodal therapy [8]. In our case, histology showed a simple lipoma.

## Conclusions

Giant lipomas in the head and neck region are rare. When they do present, careful pre-operative planning needs to be considered. This allows the exclusion of malignant lesions such as liposarcoma but also allows the surgeon to plan their operation in the context of distorted anatomy. Patients should be thoroughly informed about the risks of the operation in these cases.
